# Beyond Weight Loss: Added Benefits Could Guide the Choice of Anti-Obesity Medications

**DOI:** 10.1007/s13679-023-00502-7

**Published:** 2023-05-20

**Authors:** Valeria Guglielmi, Silvia Bettini, Paolo Sbraccia, Luca Busetto, Massimo Pellegrini, Volkan Yumuk, Anna Maria Colao, Marwan El Ghoch, Giovanna Muscogiuri

**Affiliations:** 1 Dept. of Systems Medicine, University of Rome Tor Vergata, Rome, Italy; 2 Internal Medicine Unit - Obesity Center, University Hospital Policlinico Tor Vergata, Rome, Italy; 3 Center for the Study and the Integrated Treatment of Obesity, Internal Medicine 3, Padua University Hospital, Padua, Italy; 4 Department of Biomedical, Metabolic and Neural Sciences, University of Modena and Reggio Emilia, 41121 Modena, Italy; 5 Division of Endocrinology, Metabolism & Diabetes Istanbul University Cerrahpaşa Medical Faculty, Istanbul, Türkiye; 6 Italian Centre for the Care and Well-Being of Patients With Obesity (C.I.B.O), Dipartimento Di Medicina Clinica E Chirurgia, Università Federico II, 80131 Naples, Italy; 7 Dipartimento Di Medicina Clinica E Chirurgia, Diabetologia E Andrologia, Unità Di Endocrinologia, Università Degli Studi Di Napoli Federico II, Via Sergio Pansini 5, 80131 Naples, Italy; 8 Cattedra Unesco ”Educazione Alla Salute E Allo Sviluppo Sostenibile”, University Federico II, Naples, Italy; 9 Department of Nutrition and Dietetics, Faculty of Health Sciences, Beirut Arab University, P.O. Box 11-5020, Riad El Solh, Beirut, Lebanon

**Keywords:** Anti-obesity drugs, Naltrexone/bupropion, Liraglutide, Semaglutide, Tirzepatide, Comorbidities

## Abstract

***Purpose of Review*:**

To highlight the added benefits of approved and upcoming, centrally-acting, anti-obesity drugs, focusing not only on the most common metabolic and cardiovascular effects but also on their less explored clinical benefits and drawbacks, in order to provide clinicians with a tool for more comprehensive, pharmacological management of obesity.

***Recent Findings*:**

Obesity is increasingly prevalent worldwide and has become a challenge for healthcare systems and societies. Reduced life expectancy and cardiometabolic complications are some of the consequences of this complex disease. Recent insights into the pathophysiology of obesity have led to the development of several promising pharmacologic targets, so that even more effective drugs are on the horizon. The perspective of having a wider range of treatments increases the chance to personalize therapy. This primarily has the potential to take advantage of the long-term use of anti-obesity medication for safe, effective and sustainable weight loss, and to concomitantly address obesity complications/comorbidities when already established.

***Summary*:**

The evolving scenario of the availability of anti-obesity drugs and the increasing knowledge of their added effects on obesity complications will allow clinicians to move into a new era of precision medicine.

## Introduction

Obesity is a chronic, progressive and relapsing disease, associated with decreased life expectancy and disability, depending on its duration, the magnitude of excess weight and the development of complications. It increases the incidence of type 2 diabetes (T2D) and cardiovascular disease (CVD) but also psychological, neurological, pulmonary and musculoskeletal diseases, resulting in a heavy financial burden on healthcare systems [1].

Despite major advances in diagnosis and an increased availability of anti-obesity treatment approaches, the prevalence of obesity continues to increase [2]. These data suggest that there is a lack of understanding as regards the management of obesity. Currently, medication used in Europe for obesity weight management is represented by orlistat, liraglutide and naltrexone hydrochloride (HCl)/bupropion HCl [3]. Orlistat is a pancreatic and gastric lipase inhibitor that generates an interference with lipase-catalysed breakdown and the subsequent systemic absorption of around 30% of dietary ingested fats [4, 5]. Liraglutide is a glucagon-like peptide-1 receptor agonist (GLP-1RA) and appetite suppressant, that acts centrally on the arcuate nucleus in the hypothalamus to suppress appetite and potentiate satiety [5–8]. Moreover, the main mechanism of action of naltrexone/bupropion relates to appetite suppression [9].

Safety and efficacy in relation to weight loss was also demonstrated in the case of new drugs, such as semaglutide, a GLP-1 RA [10], cagrilintide, a long-acting acylated amylin analogue [11••, 12••] and tirzepatide, a combination of the gastric inhibitory polypeptide (GIP) receptor with GLP-1RA [13].

Despite the availability of these drugs for the treatment of obesity, there are no guidelines specifying which therapeutic intervention should be used, based on patients’ comorbidities. Since obesity is a remarkably heterogeneous disease, it could also be expected that the heterogeneity among patients with obesity results in a different weight loss response to obesity interventions, such as diets, medication, devices and surgery.

Recently, a phenotype-guided approach for the treatment of obesity, based on measurable components of food intake and energy expenditure has been proven to be associated with greater weight loss; a higher proportion of patients lost more than 10% after one year, as compared with patients whose treatment was not phenotype-guided [14•].

Therefore, by moving a step closer towards a precision medicine and in order to optimize obesity treatment, we have questioned whether an obesity complications-guided approach, based on the evidence from preclinical and clinical studies, can be of help in individualizing obesity treatment.

**Fig. 1:**
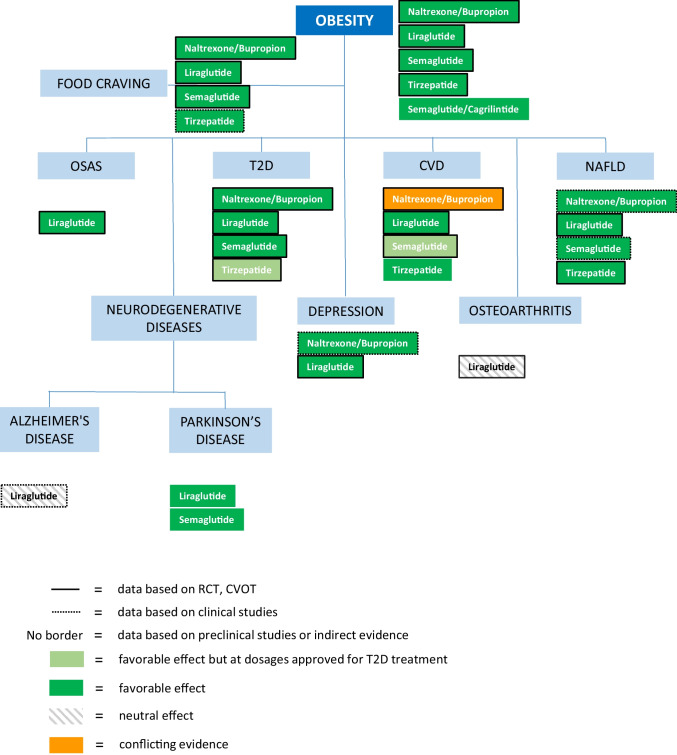
Anti-obesity drugs and their effects on obesity complications or comorbidities, based on randomized clinical trials (RCT), cardiovascular outcome trials (CVOT) for CV disease, clinical and preclinical studies or indirect evidence. The data refer to dosages approved for the treatment of obesity, unless indicated otherwise (e.g., in light green). Should no data be available, the name of the drug is omitted

Thus, the aim of this manuscript is to review the current evidence on centrally-acting, anti-obesity drugs, both already approved and those which are upcoming, and to consider obesity-related, conventional comorbidities, as well as the added benefits, in order to provide tools for a more precise and patient-tailored treatment, potentially resulting in greater effectiveness (Fig. 1; Table 1).

**Table 1:** Benefits of anti-obesity drugs on obesity comorbidities*

Anti-obesity drugs	Comorbidities – main studies
Naltrexone/Bupropion	**Depression** ( *McElroy SL *et al*. 2013 *[177]*)* **Type 2 diabetes** (*Hollander *et al*. 2013 *[75]*)* **Non-alcoholic fatty liver disease** *( Bajaj HS *et al*. 2021 *[115]*)*
Liraglutide	**Depression** (*Mansur RB *et al*. 2017 *[183]*)* **Type 2 diabetes** (*Madsbad S. *et al*. 2009 *[80]*)* **Non-alcoholic fatty liver disease** (*Armstrong MJ *et al*. 2016 *[122]*)* **Obstructive Sleep Apnea Syndrome** (*Blackman A *et al*. 2016 *[144]*)* **Cardiovascular diseases** *(Marso SP *et al*. 2016 *[100]*)*
Semaglutide	**Type 2 diabetes** *(Sorli C *et al*. 2017 *[85]*)* **Cardiovascular diseases** *(Husain M *et al*. *[107]*)* **Non-alcoholic fatty liver disease** *(Newsome PN *et al*. 2021 *[125]*)*
Tirzepatide	**Type 2 diabetes** (*Frias JP *et al*. 2018 *[50]) **Cardiovascular diseases** (*Dahl D *et al*. 2022 *[97]*)* **Non-alcoholic fatty liver disease** (*Gastaldelli A *et al*. 2022 *[134]*)*

^*^Direct evidence deriving from clinical studies (i.e. RCT, observational studies etc.)

## Approved and Upcoming, Centrally-Acting, Anti-Obesity Drugs

### Naltrexone/Bupropion

is a drug, licensed as an adjunct to lifestyle changes, such as a low-calorie diet and regular physical activity and it is used for the management of weight in adults with obesity (Body Mass Index, BMI ≥ 30 kg/m^2^) or overweight (with a BMI ≥ 27 kg/m^2^) with at least one weight-related comorbidity [9].

Contained in each prolonged-release tablet is 8 mg of naltrexone hydrochloride and 90 mg of bupropion hydrochloride. The initial dose is one tablet daily, which should be increased over a four-week period to the recommended dose of two tablets twice daily (32 mg naltrexone and 360 mg bupropion per day). The suggestion is to assess weight loss after 16 weeks of treatment; the drug should be discontinued if at least 5% weight loss has not been achieved [9, 15].

Bupropion, originally developed as an antidepressant, acts by inhibiting dopamine and noradrenaline reuptake, while naltrexone is an opioid antagonist [16].

The main mechanism of action of naltrexone/bupropion relates to appetite suppression [9]. It has been suggested that naltrexone/bupropion exerts a synergistic effect on the hunger centre located in the hypothalamus, reducing appetite and the impact of food cues, and increasing activation of the regions of the brain responsible for self-control [17]. The most common side effect was nausea [18].

The efficacy of naltrexone/bupropion on obesity was mainly demonstrated in four randomized, double-blind, placebo-controlled, 56-week, phase-3 clinical trials, with 4536 adult subjects: COR-I, COR-II, COR-BMOD and COR-DM. COR-I and II trials were ad hoc designed trials, with weight change as the primary endpoint. In the COR-I study, 1742 subjects were enrolled and were randomized in a 1:1:1 ratio to naltrexone HCl 32 mg/bupropion HCl 360 mg, n = 583; naltrexone HCl 16 mg/bupropion HCl 360 mg, n = 578; placebo, n = 581. Half of the subjects (n = 870) completed 56 weeks of treatment (n = 296; n = 284; n = 290, respectively) and 83% (n = 1453) were included in the analysis [19]. The mean percentage change in body weight was -1.3% in the placebo group, while it was -6.1% in the naltrexone 32/bupropion 360 group (p < 0.0001 vs. placebo) and -5.0% in the naltrexone 16/bupropion 360 group (p < 0.0001 vs. placebo). Sixteen percent of subjects in the placebo group had a reduction in body weight of 5% or more, versus 48% in the naltrexone 32/bupropion 360 group and 39% in the naltrexone 16/ bupropion 360 group (p < 0.0001 vs. placebo) [19]. In the COR-II study, 1496 subjects were enrolled and were randomly divided 2:1 into naltrexone HCl 32 mg/bupropion HCl 360 mg (NB32) or placebo for up to 56 weeks. The primary end points were weight change and percentage of patients reaching ≥ 5% weight loss at week 28 [20]. Significantly greater weight loss was reported in the intervention group versus the placebo group at week 28 (-6.5 vs. -1.9%; p < 0.001) and week 56 (-6.4 vs. -1.2%; p < 0.001). A higher percentage of NB32-treated subjects achieved ≥ 5% weight loss versus those in the placebo group at week 28 (55.6 vs. 17.5%; p < 0.001) and week 56 (50.5 vs. 17.1%; p < 0.001) [20].

### Liraglutide

is a glucagon-like peptide-1 (GLP-1) analogue with a 97% homology to human glucagon-like peptide-1 [5–7], due to molecular modifications [8]. GLP-1 is secreted by the L-cells of the gastrointestinal tract when nutrients arrive in the lumen. It produces glucose-dependent insulin secretion, a decrease in plasma glucagon concentrations, delayed gastric emptying and appetite suppression [8]. Since GLP-1 receptor agonists stimulate insulin release and inhibit glucagon secretion in a glucose-dependent fashion, there is a low risk of hypoglycaemia [3]. Endogenous GLP-1 has a half-life of 1.5–2 min, as it is degraded by dipeptidyl peptidase 4 (DPP-4) and neutral endopeptidases (NEP). Liraglutide, conversely, is relatively resistant to DPP- 4 degradation and has a plasma half-life of 11–13 h after subcutaneous administration, providing 24-h duration of action [21, 22]. Lastly, liraglutide does not interfere with the cytochrome P450 system and it is eliminated as metabolites in urine and faeces [22]. Saxenda®, liraglutide 3,0 mg, was approved in 2014 by the US Food and Drug Administration (FDA) [3] and in April 2015 by the European Medicines Agency (EMA) («Saxenda—EPAR summary for the public» 2015) for the treatment of obesity. The guidelines of the European Association for the Study of Obesity (EASO) recommend the use of pharmacologic therapy in adults who have a BMI ≥ 30 kg/m^2^ or ≥ 27 kg/m^2^ with at least one weight-related comorbid condition (e.g., hypertension, dyslipidaemia, insulin resistance, T2D) [23].

The most frequent adverse events are nausea and vomiting, which mainly occur during the first few weeks of treatment and are usually tolerable, not leading to the discontinuation of therapy; other adverse effects include diarrhoea, constipation, dyspepsia and abdominal pain [24–27]. The risk of acute pancreatitis has been reported in a small percentage of treated patients [6, 22].

It was demonstrated that liraglutide causes thyroid C-cell tumours in mice, however, there is no evidence to support the fact that liraglutide causes such tumours in humans [24]. Liraglutide does not interfere with other medication and does not affect the absorption of oral medication. There are currently no dosage adjustments provided for the use of liraglutide in the presence of renal or hepatic dysfunction, however, caution should be used. Pregnant patients should avoid the use of liraglutide [22].

The dose of liraglutide should be titrated by 0.6 mg weekly, up to a 3.0 mg daily dose. If the dose escalation is not tolerated due to adverse gastrointestinal effects, delaying the titration by one week may be considered. Patients should be evaluated after 16 weeks: if the patient has not lost at least 4% of his or her baseline body weight, liraglutide should be discontinued [22].

The SCALE (Satiety and Clinical Adiposity – Liraglutide Evidence) Obesity study was a 56-week, double-blind, randomized controlled trial (RCT) involving 3731 patients without T2D and with a body-mass index of ≥ 30 kg/m^2^ or ≥ 27 kg/m^2^ and with dyslipidaemia or hypertension [6]. The coprimary endpoints were weight change from the baseline and, in particular, a loss ≥ 5% and ≥ 10% of their baseline body weight at week 56. After 56 weeks, patients in the liraglutide group had lost a mean of 8.4 ± 7.3 kg of body weight, and those in the placebo group had lost a mean of 2.8 ± 6.5 kg. Patients in the liraglutide group lost at least 5% of their body weight, compared to the placebo group (63.2% vs. 27.1%), more than 10% of their body weight (33.1% vs. 10.6%), and more than 15% of their body weight (14.4% vs. 3.5%) [6]. There were no differences in the treatment effects or safety profile of 3.0 mg of liraglutide for individuals with a BMI of 27 to < 35 or ≥ 35 kg/m^2^, and its effects and safety profile were consistent across racial subgroups [28]. The efficacy of liraglutide in terms of causing weight loss among patients with T2D was also demonstrated [26]. Recent clinical trials and metanalyses investigated the use of liraglutide in adolescents (> 12 years old) suffering from obesity, as it has demonstrated a significantly greater reduction in the BMI standard-deviation score than the placebo plus lifestyle therapy [29•, 30]). The existing literature offers some case reports of successful treatment with liraglutide in subjects with compulsive, food-related behaviour, associated with autism [31] and hypothalamic obesity [32]; some encouraging results have been noted in patients with obesity and severe mental illness [33].

### Semaglutide

is a GLP-1RA, with 94% homology with native human GLP-1 and structural modifications that allow for reversible albumin binding, reduced renal clearance and degradation by DPP-4, resulting in a longer half-life (155 to 184 h) [10, 34]. As with other GLP-1RAs, the most common side effects of semaglutide are gastrointestinal issues, mostly mild-to-moderate in severity, transient and occurring during dose escalation [35]. Although semaglutide increases the risk of biliary disease (primarily cholelithiasis), no unexpected safety issues have arisen to date, therefore, the established safety profile is similar to that of other GLP-1RAs.

Semaglutide was approved in 2021 by the FDA and EMA as an adjunct to reduced calorie intake and increased physical activity for chronic weight management in adults with obesity (BMI ≥ 30 kg/m^2^) or overweight (BMI ≥ 27 kg/m^2^) with at least one weight-related comorbidity [36, 37]. The semaglutide-weight loss was obtained by limiting food intake with minimal effects on energy expenditure [38]. Despite preclinical studies reported that semaglutide had limited efficacy in crossing the blood–brain barrier, semaglutide was proven to access the brainstem, septal nucleus and hypothalamus directly [39]. Semaglutide also induces central c-Fos activation in secondary brain areas without direct GLP-1R interaction, such as the lateral parabrachial nucleus, thus having direct and indirect effects on neutral pathways involved in homeostatic (appetite, hunger, satiety) and hedonic (food preference, cravings, control of eating) aspects of food intake and reward-related behaviours pertaining to food [39]. Conversely, only a very small percentage of weight loss is explained by delayed gastric emptying and gastrointestinal side effects (nausea or vomiting) [40].

The dose of semaglutide for reaching an efficient weight loss is 2.4 mg, injected subcutaneously once-weekly [41] and is reached by increasing the initial dose of 0.25 mg at four-week intervals.

The Semaglutide Treatment Effect in People with obesity (STEP) was a pivotal, global, phase-3, clinical development programme that evaluated 2.4 mg of semaglutide once-weekly for weight management [42]. As a whole, the first four STEP trials reported reductions of approximately 15% of initial body weight after 68 weeks of treatment, associated with improvements in cardiovascular (CV) risk factors and physical functioning [42]. The STEP 5 trial assessed the durability of the drug effect and examined the trajectory of weight loss over 104 weeks, showing that the initial reduction in weight, which plateaued after approximately week 60, was maintained for the remainder of the study [43].

A placebo-controlled, phase-3 trial (NCT05035095, OASIS 1) is ongoing, to address the effects of 50 mg of oral semaglutide once-daily over a period of 68 weeks in subjects who are either overweight or with obesity [44].

### Semaglutide/Cagrilintide

Cagrilintide is a long-acting, acylated amylin analogue, with agonistic effects on both native amylin and calcitonin receptors and has been investigated for weight management. Amylin is a glucoregulatory hormone, co-secreted with insulin by pancreatic β-cells that regulate glucose homeostasis by delaying gastric emptying, suppressing postprandial glucagon release and inducing meal‐ending satiety [45]. Native amylin is involved in the regulation of appetite and satiation through the activation of receptors in the area postrema and nucleus of the solitary tract of the hindbrain [46]. In addition, the hypothalamus, ventral tegmental area and laterodorsal tegmental nucleus are targeted by amylin to influence the hedonic aspects of food intake, such as reward-guided behaviours that influence food choices and preferences [47]. Thus, although both semaglutide and cagrilintide induce satiety, while operating in different regions of the brain, their combination has the potential to act additively to improve weight control. The key point with regard to developing combination therapies is the need to be more effective than monotherapy but to maintain a reasonable price in terms of adverse events and cost.

Based on a phase-2 trial that reported dose-dependent reductions in body weight of up to 10.8% after a 26-week treatment with sc cagrilintide once-weekly [12••], a phase-1-b study tested the safety and tolerability of six multiple-ascending doses of sc cagrilintide (with doses ranging from 0.16 to 4.5 mg), administered once-weekly in combination with 2.4 mg of semaglutide to subjects, who are either overweight or with obesity. Changes in body weight, assessed as the exploratory endpoint, were as high as 17.1% (15.9 kg; with 2.4 mg of cagrilintide plus 2.4 mg of semaglutide) after 20 weeks of treatment [11••]. A RCT currently ongoing, aims to test the efficacy and safety of cagrilintide sc 2.4 mg in combination with semaglutide sc 2.4 mg (cagrisema sc 2.4 mg/2.4 mg) once-weekly in participants with overweight or obesity (REDEFINE 1, NCT05567796) [48].

### Tirzepatide

Further to the aforementioned GLP-1Ras, such as liraglutide and semaglutide, a new anti-obesity drug, administered once a week and combining the GIP receptor with GLP-1R agonism into a single novel molecule has been developed and named tirzepatide [49]. It seems that GIP is a hormone that potentiates the effects of GLP-1RAs.

The adverse drug effect of tirzepatide is similar to that seen in the GLP-1RAs [50], whereby the most common side effects associated with tirzepatide are related to gastrointestinal symptoms, i.e., diarrhoea, nausea and vomiting [13].

A large, controlled trial [51] was recently conducted and enrolled 2539 adults with overweight or obesity, who were randomly divided into four groups and received 5 mg, 10 mg or 15 mg of sc tirzepatide once-weekly or a placebo for 72 weeks. The mean weight loss percentage (WL%) at week 72 was − 15.0% with 5 mg, − 19.5% with 10-mg and − 20.9% with 15-mg weekly doses of tirzepatide, and − 3.1% with the placebo. The proportion of patients who had a WL% of 5% or more was 85%, 89%, 91% and 35% with 5 mg, 10 mg and 15 mg of tirzepatide and placebo, respectively. Moreover, more than 50% of patients in the 10 mg and 15 mg groups experienced a WL% ≥ 20% as compared with 3% in the placebo group [51]. In addition, a recent systematic review and meta-analysis confirmed that the WL% with tirzepatide is dose-dependent and this drug appears to induce a greater WL when compared to GLP-1 RAs, (up to 7.16 kg more with 15 mg of tirzepatide) [52].

### Food Cravings

Food cravings are strongly associated with increased food intake and are a significant predictor of greater weight gain over time and a lifetime high BMI [53]. Although several types of food cravings have been defined, they are not always completely dissociable. However, the type of craving may be less important than its severity in terms of contributing to increased eating. While the obesogenic food environment negatively impacts on everyone to some extent, it has been recognized that some individuals may be more sensitive to food-related cues or may experience more cravings.

#### Naltrexone/Bupropion

Naltrexone antagonism of the opioid receptors has been reported as decreasing the subjective delightfulness or liking of particular foods; in particular, naltrexone plays a role in modulating the reward aspects of eating and the consumption of energy dense sugar and fat-laden foods [54–56]. The blockade of the l-opioid receptor with naltrexone, in order to antagonize the auto-inhibitory actions of the bupropion-stimulated release of endogenous opioids, is the basis of the combination treatment with bupropion and naltrexone for obesity management. Indeed, the injection of naltrexone and bupropion into the reward system of mice resulted in a reduction of food intake, and the effect was potentiated when these were administered together. A phase-3 trial demonstrated that the synergic treatment of naltrexone and bupropion significantly reduces food reward aspects, measured by the Control of Eating Questionnaire (CoEQ) [19, 20, 57]. Thus, patients displayed reduced frequency and intensity of food cravings. A recent randomized double-blind placebo-controlled trial showed that behavioral weight loss therapy (BT) and naltrexone-bupropion were efficient for binge-eating disorder [58], with BT being superior to the absence of BT.

#### Liraglutide

acts on the central nervous system increasing satiety and hence reducing ad libitum meal intake [59]. In response to highly palatable foods, liraglutide decreases neural activation in the brain areas associated with appetite and reward [60]. To regulate food intake, our brain integrates external cues with internal state signals, such as the feeling of hunger [61–63]. Incentive motivation is defined as the process that translates an expected reward into the effort spent to obtain it and this is regulated by the dopaminergic midbrain and its mesoaccumbens dopaminergic projections [64]. GLP-1 has a modulatory role in the midbrain dopamine function, blunting the interaction effect of hunger on motivation, depending on insulin sensitivity, meaning that it can restore dysregulated motivational behaviour in insulin-resistant humans [65]. Liraglutide, combined with intensive behavioural therapy (IBT), is associated with greater short-term results in dietary disinhibition, eating disorder psychopathology and shape concern than IBT alone [59, 66].

The Binge Eating Liraglutide Intervention (BELIEVE), a phase-3 RCT, confirmed the efficacy of liraglutide compared to placebo in reducing the number of binge episodes per week, ameliorating dietary disinhibition, perceived hunger, quality of life and a depressed mood [67].

Another RCT showed a significant difference in hunger, fullness and food preoccupation up until week 24 between liraglutide + IBT and placebo + IBT: these differences were not maintained at week 52, despite the greater weight loss in the liraglutide-treated participants [68].

#### Semaglutide

Clinically, 2.4 mg of semaglutide once-weekly has been shown to decrease energy intake by reducing appetite, food cravings and a preference for fatty, energy-dense foods, as well as improving control of eating and meal portion size management [38, 40].

The severity and type of food cravings were assessed in a subgroup of participants from Canada/USA in a STEP 5 trial [69] by using the CoEQ, comprising 19 items grouped into four domains: craving control, craving for savoury food, craving for sweet food or positive mood. Among the participants receiving treatment with semaglutide (n = 88) vs. placebo (n = 86), all four domain scores significantly improved at week 20 and 52. The domains of craving control and craving for savoury food maintained their improvements up to week 104 and the individual, craving-related items which were significantly reduced were: desire to eat salty and spicy food, craving for dairy food, craving for starchy food, difficulty in resisting cravings and difficulty in controlling eating. By contrast, the scores for hunger and fullness improved significantly at week 20 only [70].

In an exploratory phase-1 trial investigating the effects of 14 mg of oral semaglutide once-daily for 12 weeks (four-week dose escalation) on the control of eating among 15 subjects with T2D, fewer and less strong food cravings, as well as better eating control (assessed by CoEQ) were reported compared to the placebo [71].

A clinical trial is underway to assess whether 50 mg of semaglutide once-daily affects food intake, hunger, satiety and food cravings among those with obesity [72].

## Type 2 Diabetes

In most cases, obesity precedes diabetes and is the most important risk factor in relation to the worldwide increase of T2D prevalence [73]; consequently, it has been increasingly recognized that many patients with T2D would benefit from having a primary weight-centric approach to diabetes treatment [74]. As such, weight loss ≥ 15% can have a disease-modifying effect among people with T2D. From this perspective, we are revising the impact of centrally-acting, anti-obesity drugs on T2D.

### Naltrexone/Bupropion

The efficacy of naltrexone/bupropion in T2D was investigated in the COR-DM trial. In this study, 505 subjects with T2D with haemoglobin A1c (HbA1c) between 7 and 10% and fasting blood glucose < 270 mg/dl (15.0 mmol/dl) [75] and taking any combination of oral diabetes medication (metformin, sulfonylureas and thiazolidinediones) were enrolled. These subjects were randomized in a 2:1 ratio to NB32 or placebo for 56 weeks and stratified by HbA1c ≤ 8% or > 8% and sulfonylurea use. Subjects in both groups followed a hypocaloric diet (a 500 kcal per day deficit, based on estimated metabolic rate) and participated in moderate physical activity (30 min of walking, five days per week). Co-primary end points were represented by the percentage weight loss from the baseline and the percentage of patients achieving ≥ 5% weight loss. Secondary end points were represented by the percentage of patients achieving ≥ 10% weight loss and changes in waist circumference, triglycerides, HDL- and LDL- cholesterol (HDL-C and LDL-C) and hs-CRP. The metabolic parameters that were followed up were HbA1c, fasting blood glucose, fasting insulin, HOMA-IR and the percentage of patients achieving HbA1c < 7.0% and < 6.5%, in addition to changes in oral antidiabetic agents.

Based on the modified intent to treat (mITT) analysis, the average weight loss was significantly higher in the intervention group versus the placebo group (-5.0 ± 0.3% vs. -1.8 ± 0.4%; p < 0.001). HbA1c reduction was higher in the intervention group versus the placebo group (-0.6 vs. -0.1%; p < 0.001) at the end of 56 weeks. In addition, a greater percentage of patients in the intervention versus the placebo group achieved HbA1c < 7.0% (44.1 vs. 26.3%) or < 6.5% HbA1c (20.7 vs. 10.2%). Statistical analysis showed that the HbA1c change was significantly correlated with weight loss in both the intervention and the placebo groups.

Changes in fasting blood glucose and fasting insulin did not significantly differ between the intervention and placebo groups. In addition, over the course of the study, fewer patients in the intervention versus the placebo group needed to increase oral antidiabetic agents, due to worsening HbA1c values [75].

A post-hoc analysis of naltrexone/bupropion versus the placebo was carried out among subjects with stable T2D on an incretin agent prior to randomization in a double-blind, placebo-controlled CV outcome trial (CVOT) [76]. After one year, the mean weight loss was significantly greater among naltrexone/bupropion patients versus placebo patients, particularly those taking DPP-4i (mean absolute difference 4.6% [p < 0.0001]) and those taking GLP-1RAs (mean absolute difference 5.2%, p < 0.0001). The rates of serious adverse effects in all areas of this analysis were lower than have been reported in other CVOTs in T2D [76].

In summary, naltrexone/bupropion has been demonstrated to be effective in reducing HbA1c and is safe among subjects with T2D, who are taking oral antidiabetic agents.

### Liraglutide

Beta-cell dysfunction is the main defect in patients with T2D, whose beta-cells number and function reduce by 50% by the time of their diagnosis [77]. Among the many ways in which the β-cell fails, there is the loss of incretin effect, which is defined as the increase in glucose-stimulated insulin secretion by intestinal insulinotropic peptides such as GIP and GLP-1. These two hormones can stimulate insulin secretion in a glucose-dependent way [78]. Liraglutide is currently approved for once-daily sc treatment of T2D at a dose of 1.8 mg [79].

The Liraglutide Effect and Action in Diabetes (LEAD™), completed in 2007, is a phase-3 development programme, divided into six clinical trials, each studying the effect of liraglutide in combination with other commonly used antidiabetic medication (LEAD 1, 2, 4, 5, 6) and in monotherapy (LEAD 3). This programme included 6500 people in 41 countries worldwide [80] and demonstrated the superiority of liraglutide compared with other antidiabetic drugs (including glimepiride, rosiglitazone, metformin, insulin glargine and exenatide) in lowering HbA1c, fasting plasma glucose and body weight [80]. A recent RCT (NCT01541215) was able to confirm that 1.8 mg of liraglutide per day (added to metformin, with or without basal insulin) improves glycaemic control in children and adolescents with T2DM, with only an increased frequency of adverse gastrointestinal events, mainly nausea [81]. Finally, and rather interestingly, liraglutide as an adjunct to insulin has been shown to improve glycaemic control, induce body weight loss and decrease exogenous insulin requirements and severe hypoglycaemia in patients with type 1 diabetes mellitus [82].

### Semaglutide

is currently approved for the once-weekly sc and once-daily oral treatment of T2D. Semaglutide acts through the incretin pathway, stimulating insulin and inhibiting glucagon secretion from the pancreatic islets in a glucose-dependent manner [83]. Subcutaneous semaglutide was evaluated in the Semaglutide Unabated Sustainability in Treatment of Type 2 Diabetes (SUSTAIN) clinical trial programme, consisting of seven randomized, controlled phase-3 trials and involving more than 8000 patients with T2D. Six efficacy trials (SUSTAIN 1–5 and SUSTAIN 7) were designed to evaluate the efficacy and safety of semaglutide vs. comparators, across the broad spectrum of T2D pharmacotherapy [84]. Semaglutide consistently reduced HbA1c and fasting plasma glucose, and self-monitored blood glucose profiles and body weight, with a lower risk of hypoglycaemia (excluding placebo and sitagliptin), demonstrating superior glycaemic control vs. a dipeptidyl peptidase-4 inhibitor (sitagliptin), other once-weekly GLP-1RAs (exenatide ER and dulaglutide) and basal insulin (IGlar) [85–90].

Oral semaglutide represents the first available oral GLP-1RA for the management of T2D. Its approval was based on the results of the randomized, phase-3a clinical trial (Peptide InnOvatioN for Early diabEtes tReatment, PIONEER) that tested oral semaglutide among patients with T2D in relation to the placebo group and active glucose-lowering medication [91]. Over periods of treatment of up to 78 weeks, oral semaglutide (7 and 14 mg once-daily) reduced HbA1c, fasting plasma glucose and body weight, thereby showing significantly greater efficacy than sitagliptin, empagliflozin, liraglutide (at week 26) and dulaglutide [91].

### Semaglutide/Cagrilintide

A phase-3 study (REDEFINE 2, NCT04982575) has been testing the efficacy of combination treatment with 2.4 mg of semaglutide plus 2.4 mg of cagrilintide in glycaemic control among subjects with overweight/obesity and T2D [92].

### Tirzepatide

The impact of tirzepatide on the glycaemic profile has been reported as fasting glycaemia, post-prandial glycaemia, HbA1c and insulin sensitivity as well as hypoglycaemia. When compared to the placebo, tirzepatide seems to exhibit a significantly higher reduction of both fasting and post-prandial glycaemia (nearly − 60 mg/dL, and nearly − 2% as HbA1c) [13, 50, 93] and improves insulin resistance by reducing the HOMA-IR index by − 0.70 [50, 94].

However, more interestingly, tirzepatide action appears to exceed other treatments, such as GLP-1 RAs and basal insulin, in lowering fasting and post-prandial glycemia by 15 to 20 mg/dL and HbA1c by nearly 1% [94–96]. Moreover, patients receiving tirzepatide had significantly lower insulin resistance, expressed as HOMA-IR, compared to GLP-1 RAs by − 0.44 [50, 94].

Finally, the occurrence of hypoglycaemia with tirzepatide did not differ from that seen in the placebo group and had a lower incidence by comparison with basal insulin [97]. However, the difference in hypoglycaemia incidences between tirzepatide and GLP-1RAs is still unclear, due to the variability in definitions of hypoglycaemia among studies [52].

## Cardiovascular Disease

Obesity is associated with an increased incidence of CV events, and weight loss achieved through lifestyle changes lowers risk factors but has no clear effect on CV outcomes. In contrast, some classes of drugs used to treat obesity exert cardioprotective effects, as shown by CVOTs in T2D patients or by indirect clinical evidence on inflammation and other CV risk factors.

### Naltrexone/Bupropion

Evidence from a randomized clinical trial, the LIGHT trial, poses the question whether naltrexone/bupropion is safe for the CV system or even decreases CV risk [98]. Indeed, this trial was prematurely interrupted by the FDA because of serious confidentiality breaches, that led to the disclosure of preliminary findings which indicated a potential positive effect on CV risk factors. The findings of the LIGHT trial, including those collected at the time, showed that 25 and 50% of the planned major CV events (MACE) (death from CV causes, nonfatal myocardial infarction, or nonfatal stroke) occurred, and when the study was interrupted, these data were published in 2016 [98]. The results at 25% MACE highlight some CV benefit from this drug, while those at 50% of MACE only demonstrated non-inferiority vs. placebo at the limit of 2.0 but not at 1.4, as hypothesized. Data at 64% MACE, when the study was closed, suggested a worse safety profile, with a higher prevalence of nonfatal stroke in the naltrexone/bupropion. However, no definite conclusion can be drawn on CV safety regarding this drug combination, since the trial was not completed and provided different results of the analyses performed at different time points.

Taken together, bupropion and naltrexone are expected to have both beneficial and detrimental CV effects. Indeed, bupropion, while possibly increasing heart rate and blood pressure by potentiating catecholaminergic neurotransmission, it exerts anti-inflammatory effects [99]. Naltrexone could blunt cardiac adaptation to ischemia, although the beneficial protective effects mediated by endogenous opioid in the endothelium, could have a positive effect on strokes and reduce toll-like, receptor4-dependent inflammation [99].

### Liraglutide

The impact of liraglutide on CV outcomes was studied in a large RCT, The Liraglutide Effect and Action in Diabetes: Evaluation of Cardiovascular Outcome Results (LEADER) trial (ClinicalTrials.gov NCT01179048) [100] including 9340 patients with advanced T2DM and high baseline CV risk. After a median follow-up of 3.8 years, patients with liraglutide exhibited a significant reduction in the first occurrence of death from CV causes, non-fatal myocardial infarction or non-fatal stroke primary outcomes, compared to patients in the placebo group, with a hazard ratio (HR) of 0.87; 95% CI 0.78–0.97, p = 0.01. Furthermore, nephropathy events were significantly lower after liraglutide therapy than placebo (HR 0.78; 95% CI 0.67–0.92), but there was no significant difference in retinopathy events [101]. Another study suggested that liraglutide did not have an impact on left ventricular systolic function compared with placebo in stable, chronic heart failure patients with and without T2D (LIVE) [102]. On the other hand, it was demonstrated that liraglutide use in subjects with heart failure and reduced left ventricular ejection fraction was implicated with an increased adverse risk of heart failure-related outcomes [103]. Since it is challenging to elucidate whether GLP-1 analogues reduce the risk of atherosclerosis independent of glycaemic control, in a mouse model of arterial hypertension, GLP-1Ras, including liraglutide, reduce blood pressure and protect endothelial function independent of glucose control. These protective effects by GLP-1Ras require the endothelial but not myeloid cells [104]. Recently, a RCT was performed to evaluate the effects of 3.0 mg of injectable liraglutide daily, plus lifestyle intervention on body fat distribution in overweight or adults with obesity with and without T2D and at high CVD risk over 40 weeks of treatment [105]. Liraglutide showed consistent effects on visceral adipose tissue (VAT), measured with MRI, across all subgroups of age, sex, race–ethnicity, BMI classification and prediabetes status at baseline, with no significant interactions observed. Therefore, it was suggested that VAT reduction may be one mechanism capable of explaining the benefits seen in CV outcomes in previous trials with liraglutide, among patients with T2D, given its different effects on glucose homeostasis, atherogenic lipids, neuromediated appetite suppression and inflammation.

### Semaglutide

The results of the SUSTAIN-6, a multicentre CVOT with a median observation time of 2.1 years, were significant for the noninferiority and superiority (although the latter was not prespecified) of semaglutide vs. placebo, reporting a 26% lower risk of the primary composite CV outcome (CV death, nonfatal myocardial infarction or nonfatal stroke) in patients treated once-weekly with 0.5 mg or 1.0 mg sc semaglutide, compared to those receiving placebo [106]. CV and all-cause mortality were also significantly reduced in the PIONEER 6 trial, which aimed to evaluate the CV safety of once-daily, oral formulations of semaglutide [107].

CV outcomes are currently being investigated among those with obesity and with established CVD (but not T2D) over 31 to 59 months of treatment in the Semaglutide Effects on Heart Disease and Stroke in Patients with Overweight or Obesity (SELECT) trial (NCT03574597) [108]. The SELECT-LIFE (NCT04972721), an observational, non-interventional survey study, will examine the long-term effects (up to 10 years) on SELECT trial participants.

### Tirzepatide

In a clinical trial that included 475 adults randomized in a 1:1:1:1 ratio to receive 5 mg (n = 116), 10 mg (n = 119) or 15 mg (n = 120) of sc injections of tirzepatide or placebo once-weekly (n = 120) over a 40-week period, one of the outcomes was changes in blood pressure [97]. At week 40, the mean reduction in systolic and diastolic blood pressure was − 6.1 to − 12.6 mm Hg and − 2.0 to − 4.5 mm Hg for the tirzepatide groups and − 1.7 mm Hg and − 2.1 mm Hg for the placebo group [97].

On the other hand, a recent meta-analysis examined the adverse CV events of tirzepatide, including seven RCTs [109]. The primary aim was to compare the time to the first confirmed occurrence of four-component, major, adverse CV events (CV death, myocardial infarction, stroke and hospitalized unstable angina) between pooled tirzepatide groups and control groups [109]. The data of 4,887 participants treated with tirzepatide and those of the 2,328 control participants were analysed. Tirzepatide did not increase the risk of major CV events in participants with T2D versus controls, revealing its safety [109].

Few studies detected changes in lipid profile, expressed as LDL-c, HDL-c and triglycerides due to tirzepatide, as compared with the placebo and GLP-1RAs. Firstly, patients receiving tirzepatide displayed significantly reduced triglycerides by nearly − 30 mg/dL and LDL‑c − 5 mg/dL, and higher HDL c blood levels by + 0.5 mg/dL, as compared to the placebo [50]. On the other hand, patients receiving tirzepatide did not have significantly different triglycerides and LDL‑c compared with GLP-1RAs (i.e., dulaglutide/semaglutide) [94], but had significantly higher HDL-c compared to dulaglutide/semaglutide + 0.72 mg/dL [50].

A recent study assessed the effect of once-weekly tirzepatide (1, 5, 10 or 15 mg), GLP-1RAs (i.e., dulaglutide) (1.5 mg) and placebo on inflammation-related biomarkers, measured at baseline, 4, 12 and 26 weeks [110]. At 26 weeks, 10 and 15 mg of tirzepatide decreased YKL-40 (also known as chitinase-3 like-protein-1), intercellular adhesion molecule 1 (ICAM-1), leptin and growth differentiation factor 15 levels vs. the baseline, as well as YKL-40 and leptin levels vs. both the placebo and GLP-1RAs. Similarly, 15 mg of tirzepatide also decreased ICAM-1 levels vs. the placebo and GLP-1RAs, as well as high-sensitivity C-reactive protein (hsCRP) levels vs. the baseline and the placebo, but not vs. GLP-1RAs. YKL-40, hsCRP and ICAM-1 levels rapidly decreased within four weeks of treatment with 10 and 15 mg of tirzepatide, whereas the decrease in leptin levels was more gradual and did not plateau by 26 weeks [110].

## Non-Alcoholic Fatty Liver Disease

Obesity is not associated only with atherogenic dyslipidaemia, hypertension and CVD, but also with metabolic complications linked to insulin resistance, such as non-alcoholic fatty liver disease (NAFLD) [111].

This term refers to a spectrum of conditions defined by the presence of approximately 5% liver fat accumulation, which progresses over time to (non-alcoholic) steatohepatitis, that might occur with or without the presence of liver fibrosis, cirrhosis and hepatocellular carcinoma [112]. A growing body of evidence supports the notion that NAFLD is a multisystem disease, representing a strong independent predictor of CV events [113], chronic kidney disease and some types of extrahepatic cancers [114]. The magnitude of this risk parallels the severity of NAFLD (especially the liver fibrosis stage) [114].

### Naltrexone/Bupropion

Naltrexone/bupropion has not been evaluated in-depth to date among patients with obesity and NAFLD, and only limited data demonstrating the hepatic effect of these medications are available. In a post-hoc analysis of four RCTs, the combination of naltrexone/bupropion for one year improved the fibrosis-4 index (FIB-4), regarded as a non-invasive index of hepatic fibrosis and independent of potential cofounders such as weight loss [115]; no change in ALT was reported [115]. Although there is scarce evidence relating to the efficacy of naltrexone/bupropion in relation to NAFLD, it could be hypothesized that the magnitude of weight loss may potentially lead to an improvement in hepatic steatosis and possibly inflammation.

### Liraglutide

Although there are no licensed treatments for NAFLD, GLP-1RAs are promising [116, 117]. Indeed, this class of drugs is beneficial for patients with NAFLD, not only by reducing the long-term risk of CVD, which is the leading cause of death in patients with NAFLD, but also by exerting hepato-protective effects [116]. Indeed, although the presence of GLP-1R in hepatocytes is still controversial [118], GLP-1RAs have been shown to improve hepatic mitochondrial function and insulin sensitivity and to inhibit endoplasmic reticulum stress. They also promote autophagy to limit free fatty acid accumulation and lipotoxicity [119].

Certain studies have demonstrated that liraglutide was able to improve liver steatosis, measured as a reduction in liver fat content in RCT [120] or in open label studies [121].

Among 52 overweight patients with biopsy-proven NASH and treated either with 1.8 mg of liraglutide daily or the placebo for 48 weeks [122], the resolution of NASH occurred mostly in patients treated with liraglutide compared with those given the placebo. Fibrosis was also less in patients with liraglutide (9% versus 36%, p = 0.04).

In a 26-week RCT, investigating the effect of antidiabetic agents on NAFLD in T2M with an inadequate glycaemic control by metformin, 75 patients were randomized (1:1:1) to receive add-on liraglutide, sitagliptin or insulin glargine. Intrahepatic lipid (by MRI-estimated proton density fat fraction, MRI-PDFF), VAT and weight decreased significantly with liraglutide (p < 0.001; p = 0.003; p = 0.005, respectively) and sitagliptin (p = 0.001; p = 0.027; p = 0.005, respectively). No significant change in MRI-PDFF, VAT or body weight was observed with insulin glargine. Subcutaneous AT decreased significantly in the liraglutide group (p = 0.020) compared to other groups. Changes in MRI-PDFF, VAT and body weight were significantly greater with liraglutide treatment than insulin glargine but did not differ significantly between liraglutide and sitagliptin (J. Yan et al. 2019). In conclusion, the improvement of NAFLD by GLP-1RA involve different pathways including, on the one hand, an adaptation of plasma insulin and glucagon concentrations with a higher insulin sensitivity and, on the other hand, a decrease in AT lipotoxicity with weight-independent mechanisms [123]. However, hepatocytes lack GLP-1 receptors [124] thus it does not seem to be a direct effect of liraglutide and its precise role has yet to be elucidated.

### Semaglutide

The effects of semaglutide, which had been reported to reduce levels of alanine aminotransferase and inflammatory markers in subjects with T2D and/or obesity in a dose-dependent manner [125], have been investigated in 67 subjects with NAFLD, using non-invasive MRI methods. After 72 weeks of treatment with 0.4 mg of semaglutide once-daily, a greater reduction in liver steatosis was achieved compared to the placebo, however, no significant changes in liver stiffness, a surrogate marker of fibrosis, were observed [126].

In patients with NASH and liver fibrosis at stage F1, F2 or F3, treatment with sc semaglutide (0.1, 0.2, 0.4 mg once-daily) for 72 weeks resulted in a higher prevalence of NASH resolution, with no worsening of fibrosis compared with the placebo [127]. However, there was no improvement in fibrosis [127], possibly due to the short trial duration, a small study population, disease heterogeneity and the intrinsic limitations of liver biopsy (e.g., sample variability and reading across pathologists) [128].

As proof of concept, the phase-2 trial (NCT03987074) has been exploring the effectiveness of 2.4 mg of semaglutide administered alone or co-administered with firsocostat and cilofexor over a 24-week period in patients with NASH and mild to moderate fibrosis. Improvements in hepatic steatosis (measured by magnetic resonance imaging proton density fat fraction), liver injury (measured by serum alanine aminotransferase) and fibrosis (assessed and measured by vibration-controlled transient elastography and enhanced liver fibrosis score) were observed with semaglutide monotherapy, with the combination regimen further improving hepatic steatosis [129].

Another placebo-controlled, phase-2 trial (NCT03987451) has been investigating the effectiveness of 2.4 mg of semaglutide in patients with NAFLD and cirrhosis for 48 weeks [130].

Lastly, a phase-3 clinical trial (NCT04822181), aiming to study the effect of sc semaglutide monotherapy once-weekly over a period of five years in patients with NASH is currently in progress. Progression to cirrhosis, improvement of inflammation and histology of ballooned hepatocytes are some of the endpoints [131].

### Semaglutide/Cagrilintide

A placebo-controlled phase-2 study (NCT05016882) has been investigating the efficacy and safety of sc semaglutide (2.4 mg once-weekly), co-administered with cagrilintide with three different doses (7.5 mg, 15 mg and 30 mg once-weekly) in subjects with NASH and fibrosis stage 2 − 4 (F2 − F4). The main endpoints are the improvement in liver fibrosis and the improvement/resolution of NASH [132]*.*

### Tirzepatide

Few studies have examined the impact of tirzepatide on NAFLDs. A post-hoc analysis of a 26-week, phase 2 study in which T2D patients were randomized to receive tirzepatide, basal insulin or placebo, tirzepatide (especially at higher doses of 10 and 15 mg/week) reduced transaminases and non-invasive indices of hepatic fibrosis (including keratin-18 and procollagen III) [133]. In line, one specific recent study [134] assessed the changes in liver fat content (LFC), measured by magnetic resonance imaging-proton density fat fraction, volume of VAT and abdominal subcutaneous adipose tissue (SAT) in response to tirzepatide or basal insulin. Exactly 296 patients were randomly assigned to treatment (tirzepatide 5 mg, n = 71; tirzepatide 10 mg, n = 79; tirzepatide 15 mg, n = 72 and basal insulin, n = 74). Baseline demographics and clinical characteristics were similar across all treatment groups. The absolute reduction in LFC at week 52 was significantly greater for the pooled tirzepatide groups with a dosage of 10 mg and 15 mg vs. the basal insulin group. Tirzepatide showed a significant reduction in LFC, VAT and SAT volumes compared with basal insulin.

A 52-week, multicenter, double-blind, placebo-controlled, phase 2 RCT investigating the efficacy and safety of tirzepatide (at 5, 10, 15 mg/week) on histological endpoints in overweight/patients with obesity and NASH is ongoing (SYNERGY-NASH; NCT04166773) [135].

## Obstructive Sleep Apnoea

Obstructive sleep apnoea (OSA) is a common, chronic, sleep-related breathing disorder, characterized by repetitive upper airway collapse during sleep, causing sleep fragmentation, oxygen desaturation and excessive daytime sleepiness. OSA is common especially in older male subjects with obesity [136]. In addition, to increased all-cause mortality, untreated OSA is associated with other adverse outcomes including CVD, cerebrovascular events, T2D and cognitive impairment [137]. OSA may also be considered as a potential influent factor of weight management outcomes, since it was found to be associated with impaired muscle energy metabolism [138], reduced levels of physical activity and exercise performance [139], as well as higher levels of ghrelin, known to be an appetite stimulant [140].

### Naltrexone/Bupropion

Currently there is no evidence relating to the efficacy and safety of naltrexone/bupropion in subjects with obesity and OSA. One crossover RCT in 12 OSA patients reported a reduction in the Apnoea-Hypopnea Index (AHI) following a single dose of naltrexone [141]. Although this is a promising result, further trials are needed in order to use naltrexone/bupropion in a safe manner in subjects with obesity and OSA.

### Liraglutide

OSA was found to be associated with a lower GLP-1 response to a glucose challenge after adjusting for gender, BMI and glycaemic status [142], whereas in severe OSA, higher fasting GLP-1 levels were found and interpreted as a compensatory mechanism [143].

The SCALE Sleep Apnoea RCT aimed to investigate whether 3.0 mg of liraglutide reduced OSA severity compared with the placebo, using the primary endpoint of change in AHI after 32 weeks, both in combination with lifestyle intervention, in participants with obesity and moderate or severe OSA, who are unable or unwilling to use CPAP treatment [144]. At week 32, the mean weight loss was significantly greater with liraglutide than with the placebo (p < 0.0001). Consistent with the greater weight loss effect, significantly greater reductions in waist and neck circumference were also observed with liraglutide compared with the placebo. In both treatment groups, most of the reduction in mean AHI occurred by week 12. At week 32, the mean reduction in AHI with liraglutide was statistically significant compared with the placebo (estimated treatment difference: − 6.1 events h − 1 (95% CI, − 11.0 to − 1.2). The treatment effect on AHI did not depend on the participants’ gender, baseline BMI or OSA severity category. However, participants with severe OSA at baseline experienced a greater mean reduction in AHI in both treatment groups. Furthermore, there was a trend which showed that more liraglutide-treated patients, rather than placebo-treated participants, tended not to meet the diagnostic criteria for OSA. At week 32, weight loss with liraglutide had not plateaued. Thus, it is possible that further weight loss-related improvement in AHI would have been possible with a longer duration of liraglutide treatment [144].

### Tirzepatide

To the best of our knowledge, there are no published results to date regarding the effect of tirzepatide on OSA, but actions have been already taken in this direction. Currently, recruitment to certain on-going clinical trials have started. For instance, the SURMOUNT-OSA study [145], initiated a few months ago, aims to evaluate the effect and safety of tirzepatide in 412 adults of both genders with OSA and obesity, randomly selected to use/not use the Positive Airway Pressure therapy, the primary outcome being the change in AHI from the baseline to the one year follow up.

## Neurodegenerative Diseases

Epidemiological evidence suggest an increased risk of developing cognitive impairment and neurodegenerative diseases among individuals with obesity or T2D [146, 147]. Insulin is an important neuroprotective growth factor and neuronal insulin resistance may underlie the link between the presence of metabolic abnormalities and both Alzheimer’s disease and Parkinson’s disease [148, 149]. In addition, obesity might be associated with neurodegeneration, by causing brain inflammation, oxidative stress, protein deposition and morphological changes in brain cells [150].

### Alzheimer’s Disease

Alzheimer's disease is the most common form of neurodegenerative dementia and is characterized by impaired memory and progressive changes in cognition, behaviour and the motor system. The main neuropathological features of Alzheimer's disease include cortical atrophy, amyloid plaques formed by aggregated amyloid β (Aβ) peptides and intracellular neurofibrillary tangles, made of hyperphosphorylated tau protein, causing a reduction in synaptic strength, synaptic loss and neurodegeneration [151]. However, the current treatments for Alzheimer's disease are symptomatic and they target neurotransmission rather than neurodegeneration or neuronal metabolism [152].

#### Liraglutide

In experimental mice models, caloric restriction has been proven to reduce Aβ and neurofibrillary tangle loads. However, the neurotrophic/neuroprotective actions of GLP-1 RAs can be considered direct actions, rather than secondary actions, due to reduced food intake [153]. Indeed, preclinical models have shown that GLP-1 can promote synaptic formation, neurogenesis and neuroprotection, and may reduce neuroinflammation [154–156]. Liraglutide was shown to attenuate impaired learning and memory, as well as synaptic plasticity, decrease hippocampal neuronal loss, reduce Aβ oligomer levels and tau hyperphosphorylation, and promote neurogenesis and homeostatic autophagy in preclinical studies [156]. The use of 1.8 mg of liraglutide, used as a treatment in Alzheimer's disease patients with a long-standing disease, prevents the expected decline of cerebral glucose metabolism that reflects disease progression, but studies up until now have not shown any differences between the groups treated with liraglutide and the placebo regarding amyloid deposition or cognition [152]. The Evaluating Liraglutide in Alzheimer's Disease (ELAD, NCT01843075) is an ongoing multi-centre, phase-2b RCT using liraglutide in patients with mild Alzheimer's dementia, with the aim of assessing the change in the cerebral glucose metabolic rate as its primary outcome [157].

#### Semaglutide

GLP-1 not only enhances insulin signalling in the brain but, since GLP-1Rs are expressed in the brain [158], the former also seems to influence the control of synaptic plasticity, thus exerting neuroprotective effects [159, 160].

In human neuroblastoma (SH-SY5Y) cell lines, semaglutide exerted neuroprotective effects against extracellular amyloid-β plaques, by enhancing autophagy and inhibiting apoptosis [161]. Preclinical models, real-world evidence studies and the post-hoc analysis of data from large CV outcome trials prompted the testing of oral semaglutide in Alzheimer’s patients [162]. Indeed, two placebo-controlled phase-3 trials (EVOKE NCT04777396 and EVOKE Plus NCT04777409) have been investigating the efficacy of oral semaglutide (once-daily 14 mg) in patients with early Alzheimer’s disease. Change in the clinical dementia rating, time of progression to dementia and the change in the Alzheimer's Disease Composite Score are some of the outcomes that will be assessed [163].

### Parkinson’s Disease

Parkinson’s disease is a progressive nervous system disorder the aetiology of which remains unclear, although genetic and environmental factors seem to be involved. The clinical hallmark of Parkinson’s disease is a motor syndrome characterized by bradykinesia, rest tremor, rigidity postural instability and gait difficulties, due to nigral degeneration and striatal dopamine depletion. Parkinson’s disease is also associated with a variety of non-motor symptoms (e.g., constipation, urinary dysfunction, orthostatic hypotension, memory loss, depression and sleep disturbances) which are likely to be related to the neurodegeneration of other structures, including the peripheral autonomic nervous system [164]. Loss of dopaminergic neurons by apoptosis or autophagy and intracellular accumulation of misfolded α-synuclein (protein involved in dopamine metabolism) in abnormal aggregates, termed Lewy bodies, are the main pathological features of the disease [165].

#### Liraglutide

GLP1-RAs not only have a protective role in relation to toxic insults, but are also able to elevate endogenous TH levels in dopaminergic neurons [166, 167]. GLP1-RAs are able to change the microglial phenotype from a pro-inflammatory to a quiescent state; moreover, microglial cells generate additional GLP-1 in their anti-inflammatory state both as a potential trophic and as an anti-inflammatory factor [149, 167]. Preclinical studies showed that liraglutide reverses nigral neuronal loss, decreases pro-inflammatory cytokines and increases striatal dopamine, nigral glial cell line-derived neurotrophic factors and TH + cells. As a result, the pro-apoptotic environment in nigrostriatal tissues reduces significantly [168]. Long-term treatment with liraglutide can protect against motor function decay and dopaminergic neuron loss [169].

An ongoing phase-2, single centre, double-blind, placebo-controlled clinical trial is testing the efficacy and safety of liraglutide in the treatment of patients with idiopathic Parkinson’s disease [170].

#### Semaglutide

In the methyl-4-phenyl-1,2,3,6-tetrahydropyridine (MPTP) mouse model of Parkinson’s disease, semaglutide (25 nmol/kg once-daily) improved most of the neuropathological features of Parkinson’s disease, reducing motor impairment, oxidative damage, inflammation and apoptosis in the substantia nigra [171]. It also increased dopamine levels by supplementing tyrosine hydroxylase levels in the brain and protected dopaminergic neurons, by reducing the aggregation of α-synuclein and enhancing the expression of “glial cell line-derived neurotrophic factor” (GDNP) [172].

A phase-2 placebo-controlled clinical trial aiming to explore the effects of 1.0 mg of sc semaglutide once-weekly on Parkinson’s disease was registered by Oslo University Hospital in 2018, but recruitment has yet to start [173]. This study was designed to assess the neuroprotective and anti-inflammatory effects of semaglutide on both motor and non-motor symptoms, nigro-striatal degeneration, cognitive function and quality of life.

## Depression

Depression is a mood disorder with a high prevalence of coexistence with many other diseases including metabolic disorders, such as obesity and T2D. Obesity increases the risk of depression and, conversely, depressive disorders are predictive of the development of obesity [174], therefore, a bidirectional relationship with a weight-related gradient has been described [175]. In addition to modulating eating behaviour, centrally-acting, anti-obesity drugs are also likely to alter emotional behaviour, due to the high expression of their receptors within the fronto-striatal and limbic circuitry.

### Naltrexone/Bupropion

may be a particularly appealing option for weight management among those with depression, as one of its components, bupropion, is an agent which has been used as an antidepressant indicator for almost 40 years. A post-hoc analysis of five randomized clinical trials (duration range, 24–56 weeks) evaluating adverse effects on mood related to the use of naltrexone/bupropion in subjects with overweight or obesity as well as with or without clinical depression at baseline found a lower incidence of depression among naltrexone/bupropion-treated patients, compared to the placebo [176]. In a small 24-week pilot study that evaluated naltrexone/bupropion effects in 25 female patients affected by a major depressive disorder, the treatment was associated with a significant reduction of Montgomery–Åsberg Depression Rating Scale (MADRS) scores, improving depressive symptoms and a reduction of body weight in these patients [177]. Moreover, in patients with obesity who are on antidepressants, naltrexone/bupropion is well tolerated and is effective in promoting weight loss regardless of antidepressant use [178].

### Liraglutide

Although GLP-1RAs seem to have a beneficial influence in terms of reversing many maladaptive brain changes relevant to depression, at the same time, they are reported to have certain unfavourable effects, such as activation of the hypothalamic–pituitary–adrenal axis and the intensification of anxiety-like behaviour in some animal studies [179]. Liraglutide has been studied in relation to depression and mood disorders, mainly for safety reasons, but with some important outcomes: not only is there no cause for concern regarding the neuropsychiatric safety of treatment with 3.0 mg of liraglutide, but phase-3 trials have shown an identical rate of suicidal ideation between the placebo and treatment groups [180]. Regarding its possible use as an antidepressant, there are some interesting behavioural studies showing that liraglutide attenuates depressive- and anxiety-like behaviours in the corticosterone induced depression mouse model via improving hippocampal neural plasticity [181, 182]. A recent study involving adults with mood disorders using the Trail Making Test-B to measure executive function and MRI for a morphometry assessment demonstrated a cognitive improvement following liraglutide administration, associated with changes in brain volumes, including caudate, putamen, middle frontal cortex, superior frontal cortex and lateral orbitofrontal cortex. These findings underscore the relationship between weight, brain structure and function [183].

## Osteoarthritis

Osteoarthritis is the most common degenerative joint condition and is the leading cause of disability. Obesity, together with ageing, remains the most important risk factor as regards the occurrence and progression of osteoarthritis (OA) not only by overloading the joints with the consequent destruction of articular cartilage, but also by alterations in innate and adaptive immune responses that could lead to inflammatory cell recruitment into the synovial joints [184].

### Liraglutide

Considering the overlap between OA risk factors and metabolic derangements, as well as the anti-inflammatory properties of GLP-1 and the expression of GLP-1R in joint tissues [185], GLP-1RAs may be considered as therapeutic candidates for managing OA. A double-blind, placebo-controlled RCT (LOSEIT) assessed that in patients with overweight or obesity and knee OA, liraglutide in adjunct to an eight-week diet induced significant weight loss but did not reduce knee pain compared to the placebo [186]. Treatment with a GLP-1RA was shown to increase bone formation and prevent bone loss after weight loss [187]. An in-vitro study on human chondrocytes, exposed to liraglutide, demonstrated that this may exert a powerful antioxidant effect in the context of OA, reducing proinflammatory cytokines and mediating cartilage degradation [188]. Some animal studies showed that an intra-articular injection of liraglutide alleviated pain-related behaviour in an in-vivo OA mouse model [189].

### Semaglutide

A placebo-controlled phase-3 trial (NCT05064735) has started to investigate the efficacy of sc semaglutide (2.4 mg, once-weekly) over a 68-week period in patients with obesity and a clinical diagnosis of knee OA in terms of change in WOMAC pain, physical function and stiffness score [190].

## Conclusions

Obesity still represents a growing worldwide health challenge. Shortened life expectancy, increased healthcare costs and cardiometabolic complications are some of the consequences of this complex disease.

Over the last few years, substantial advances in the understanding of the determinants, pathophysiology, assessment and treatment of obesity have been made. Although bariatric surgery still represents the most effective therapeutical approach, with well-established benefits, pharmacotherapy has been enriched with new and more effective, centrally-acting drugs, showing promise for even greater weight loss efficacy that could revolutionize obesity management in the years to come. The perspective of having a wider range of treatments increases the interest in their putative, pleiotropic effects and the chance to tailor therapy. In this review, we highlight the added benefits of these approved and upcoming, centrally-acting, anti-obesity drugs, focusing not only on the most common metabolic and CV effects but also on their less explored clinical benefits and drawbacks. The evolving scenario of the availability of anti-obesity drugs and the increasing knowledge of their added effects on obesity complications will allow clinicians to move into a new era of precision medicine.

## Declarations

### Employment

No employment to declare.
